# Prediction of metabolite–protein interactions based on integration of machine learning and constraint-based modeling

**DOI:** 10.1093/bioadv/vbad098

**Published:** 2023-07-17

**Authors:** Fayaz Soleymani Babadi, Zahra Razaghi-Moghadam, Fatemeh Zare-Mirakabad, Zoran Nikoloski

**Affiliations:** Departement of Mathematics and Computer Science, Amirkabir University of Technology, Tehran, Iran; Systems Biology and Mathematical Biology, Max Planck Institute of Molecular Plant Physiology, Potsdam, Germany; Departement of Mathematics and Computer Science, Amirkabir University of Technology, Tehran, Iran; Systems Biology and Mathematical Biology, Max Planck Institute of Molecular Plant Physiology, Potsdam, Germany

## Abstract

**Motivation:**

Metabolite–protein interactions play an important role in regulating protein functions and metabolism. Yet, predictions of metabolite–protein interactions using genome-scale metabolic networks are lacking. Here, we fill this gap by presenting a computational framework, termed SARTRE, that employs features corresponding to shadow prices determined in the context of flux variability analysis to predict metabolite–protein interactions using supervised machine learning.

**Results:**

By using gold standards for metabolite–protein interactomes and well-curated genome-scale metabolic models of *Escherichia coli* and *Saccharomyces cerevisiae*, we found that the implementation of SARTRE with random forest classifiers accurately predicts metabolite–protein interactions, supported by an average area under the receiver operating curve of 0.86 and 0.85, respectively. Ranking of features based on their importance for classification demonstrated the key role of shadow prices in predicting metabolite–protein interactions. The quality of predictions is further supported by the excellent agreement of the organism-specific classifiers on unseen interactions shared between the two model organisms. Further, predictions from SARTRE are highly competitive against those obtained from a recent deep-learning approach relying on a variety of protein and metabolite features. Together, these findings show that features extracted from constraint-based analyses of metabolic networks pave the way for understanding the functional roles of the interactions between proteins and small molecules.

**Availability and implementation:**

https://github.com/fayazsoleymani/SARTRE.

## 1 Introduction

Small molecules exert their regulatory role by binding to proteins (Li *et al.* 2010, Li and Snyder 2011). The regulation of activity of proteins, ranging from enzymes and transporters to transcription factors and structural proteins, by small molecules represents an evolutionary conserved mechanism that facilitate organismal responses to developmental and environmental cues (Piazza *et al.* 2018). The best-studied example of a metabolite–protein interaction (MPI) network is that of competitive or allosteric regulation of enzyme activities by small molecules that are similar with either the substrate or the product of the catalyzed reaction (Link *et al.* 2013, Alam *et al.* 2017, Diether and Sauer 2017, Reznik *et al.* 2017, Razaghi-Moghadam *et al.* 2021). Yet, there are gaps in our knowledge regarding understanding of the functional role of MPIs in the context of metabolic networks and the usage of genome-scale metabolic networks across different organisms (Palsson and Yurkovich 2022) to address this problem.

MPI networks for different biological systems have been assembled by applying a variety of high-throughput *in vitro* and *in vivo* approaches (Orsak *et al.* 2012, Link *et al.* 2013, Piazza *et al.* 2018, Diether *et al.* 2019, Luzarowski *et al.* 2021). The resulting datasets along with the MPIs, available from different databases [e.g. BRENDA (Scheer *et al.* 2011) and STITCH (Kuhn *et al.* 2008)], allow us to search for patterns and functional roles of MPIs in metabolic networks. For instance, mining of the existing MPI networks has led to the finding that: (i) inhibitory MPIs are the most prevalent, with domination of competitive inhibition (Alam *et al.* 2017, Reznik *et al.* 2017); (ii) the competitive and allosteric inhibitory interactions are largely due to structural similarity between the substrate and competitive inhibitor; hence, they are found in the network vicinity of the regulated enzyme (Alam *et al.* 2017, Reznik *et al.* 2017); and (iii) MPIs are non-randomly distributed in the network, but the pattern cannot be explained by thermodynamics principles (Reznik *et al.* 2017).

The assembled MPI networks have also been used for prediction of new MPIs using two sets of complementary approaches: (i) machine/deep learning based on structural and ontology-based features and (ii) learning of MPIs by considering the effects of metabolites on fluxes of metabolic reactions. With respect to the first category, advances in deep learning have resulted in plethora of approaches to predict. presence/absence of interaction, binding affinity, and interaction sites [for a review, see Zhao *et al.* (2022)]. For instance, the assembled MPI networks have already been used with deep neural networks to predict MPIs for 23 metabolites and 9631 proteins in four species (Zhao *et al.* 2021). In addition, supervised machine learning using fingerprints and metabolite participation in different reactions has been used to predict competitive inhibitory MPIs (Razaghi-Moghadam *et al.* 2021). All these predictions rely on extraction of features that represent molecular structure, protein sequence, secondary structure of proteins, or ontology/network-related terms assigned to proteins. In the second category are approaches that predict the effect of metabolite on the flux of a metabolic reaction by fitting different enzyme kinetic forms to data of predicted and/or estimated flux, using the constraint-based modeling network, relative and absolute quantification of metabolite levels as well as absolute quantification of protein abundances (Hackett *et al.* 2016). The approaches in the second class are more resource demanding, as they rely on omics data, but are more versatile due to the ability to consider, compare, and discriminate several possible modes of regulation. They also provide insights into the functional roles of MPI on regulation of reaction and pathway flux. In contrast, the approaches in the first class do not yet facilitate characterization of the functional role of MPIs.

Against this background, here, we explore the extent to which features extracted from analysis of metabolic networks can be used in prediction of MPIs via supervised machine-learning approaches. In such a way, we aim to capitalize on the available gold standards of MPIs for model organisms, while making use of the advances in constraint-based metabolic modeling (Bordbar *et al.* 2014). Flux balance analysis (FBA) (Varma and Palsson 1994), as the principle representative of this modeling framework, has been very successful in predicting diverse cellular phenotypes related to metabolic fluxes and growth by forgoing the relation between flux and metabolite concentration and relying on linear programming (LP) formulation. Further, thermodynamic metabolic flux analysis (Henry *et al.* 2007) and extensions thereof (Akbari *et al.* 2021) have also allowed the prediction of metabolite concentration ranges at the cost of increasing the computational complexity of the optimization problems. Another approach, termed flux imbalance analysis (Reznik *et al.* 2013), has used shadow prices—the variables in the problem dual to the LP formulation of FBA—to investigate their relation to growth limitation as well as to the temporal variation in the concentration of intracellular metabolites. In these analyses, shadow prices characterized the effect of metabolite deviation from steady state on the objective of maximizing the flux through the biomass reaction, modeling growth.

Unlike flux imbalance analysis, here, we use the concept of shadow prices in the context of flux variability analysis (FVA) to devise features of metabolite–reaction pairs that we then combine with information from gene–protein–reaction (GPR) rules as input to supervised learning of MPIs. We term the resulting framework SARTRE, for **s**h**a**dow p**r**ice-based me**t**abolite–p**r**otein int**e**raction, and test its performance with different species-specific gold standards and genome-scale metabolic networks of *Escherichia coli* and *Saccharomyces cerevisiae*. We focused on the two model species due to the curated metabolic network models and gold standards of MPIs that can be used in development of models using supervised learning approaches. In addition, we investigate to what extent the usage of the shadow price-based features contributes to the prediction of MPIs.

## 2 Methods

### 2.1 Constraint-based modeling of metabolism

Stoichiometric genome-scale metabolic models (GEMs) have been developed for many organisms (Maranas and Zomorrodi 2016). Central to these models is the stoichiometric matrix $S_{m\times r}$ depicting the metabolic reactions. In the matrix S, the rowi refers to the metabolite $M_{i}\in M$, which $M=\{M_{1},M_{2},\ldots ,M_{m}\}$ is the set of metabolites, while the column j denotes the reaction $R_{j}$ from the set of reactions $R=\{R_{1},R_{2},\ldots ,R_{r}\}$. The value $S_{ij}$ represents the stoichiometric coefficient with which metabolite $M_{i}$ participates in the reaction $R_{j}$ (Orth *et al.* 2010). Genes encoding respective enzymes that catalyze reactions can be described by GPR rules. GPRs in GEM categorize into four classes: one-to-one, isozymes, and multiunit protein complexes (Maranas and Zomorrodi 2016). We define the set of existing proteins in the model as $P=\{P_{1},P_{2},\ldots ,P_{n}\}$, and each protein $P_{k}\in P$ is represented by a vector named $\Phi_{k}^{P}$, $|\Phi_{k}^{P}|=r$, as follows:


(1) $$\Phi_{kj}^{P}=\{\begin{array}{cc} 1\;\; & P_{k}\;\text{participates in the GPR rule of reaction}\;j,\\ 0\;\; & \text{otherwise}. \end{array}$$

### 2.2 Primal and dual formulations of FBA

Stoichiometric information, along with the steady-state assumption, leads to linear equations to reaction fluxes in a metabolic network. FBA is a constraint-based method to determine the flux distribution of reactions by solving a linear optimization problem that maximizes the flux through the biomass reaction, i.e. the specific growth rate.

The primal LP formulation of FBA can be written as follows (Reznik *et al.* 2013): where


(2) $$\begin{array}{c} \max\;Z=c^{T}v,\\ s.t.\;\;\mathrm{Sv}=b,\\ v^{\mathrm{LB}}\leq v\leq v^{\mathrm{UB}}, \end{array}$$

-
S is the stoichiometric matrix.
-
$v=[v_{1},v_{2},\ldots ,v_{r}]$ is the variable vector of metabolic fluxes.
-
$v^{\mathrm{LB}}=[v_{1}^{LB},v_{2}^{LB},\ldots ,v_{r}^{LB}]$ is a vector of lower bounds for fluxes.
-
$v^{\mathrm{UB}}=[v_{1}^{UB},v_{2}^{UB},\ldots ,v_{r}^{UB}]$ is a vector of upper bounds for fluxes.
-
$b=[b_{1},b_{2},\ldots ,b_{m}]$ is a vector of production/consumption of metabolites. Based on the steady-state assumption $b_{i}=0$ for each i∈{1,2,…,m}.
-
$c=[c_{1},c_{2},\ldots ,c_{r}]$ is a vector that defines the coefficient of fluxes in the objective function.

Every LP problem has a dual LP problem. To define the dual problem, we consider dual variables for every constraint in primal problem Equation (2). For the equality constraints, vector $\lambda =[\lambda_{1},\lambda_{2},\ldots ,\lambda_{m}]$ is defined. For the inequality constraints, we introduce vectors $q_{1}$ and $q_{2}$ for the lower and upper bound of flux v, respectively (Orth *et al.* 2010). As a result, the dual problem is obtained as follows:


(3) $$\begin{array}{c} \min\;Z=\lambda^{T}b-q_{1}^{T}v^{\mathrm{LB}}+q_{2}^{T}v^{\mathrm{UB}}\\ s.t.,\;c^{T}=\lambda^{T}S+q_{1}^{T}+q_{2}^{T},\\ q_{1},q_{2}\geq 0. \end{array}$$

By solving Equation (3), vector λ, that contains shadow prices, is obtained. The value $\lambda_{i}$ indicates the changes in the objective function Z if $b_{i}$ [the right-hand-side of the problem in Equation (2)] is increased by one unit (Winston and Goldberg 2004).

### 2.3 Tackling degeneracy of FBA

We examined the degeneracy of the FBA problem by maximizing the flux of each reaction separately. In their basic feasible solution, there is at least one basic variable equal to zero. This can be used to conclude that the FBA problems are degenerate. Therefore, we assess two types of uncertainties on each shadow price $\lambda_{i_{\mathrm{test}}}$, where $i_{\mathrm{test}}\in \{1,2,\ldots ,m\}$, namely:

- Infeasibility, whereby at least one of the values for maximum allowable decrease ($G_{i_{\mathrm{test}}}^{-}$) and maximum allowable increase ($G_{i_{\mathrm{test}}}^{+}$) for $b_{i_{\mathrm{test}}}$ of problem in Equation (2) is equal to zero. These values are defined as the maximum range in which the basis remains unchanged.
- Two-sided, whereby increasing and decreasing $b_{i_{\mathrm{test}}}$ of problem in Equation (2) results in different changes in the objective function.

The calculated shadow prices are valid if it satisfies the following two conditions: first,


(4) $$G_{i_{\mathrm{test}}}^{+}\neq 0\;\wedge \;G_{i_{\mathrm{test}}}^{-}\neq 0.$$

Second, we solve two other LP problems, given by Equations (5) and (6). These problems are based on FBA [problem in Equation (2)], except that steady-state assumption is positively and negatively perturbed for constraint $i_{\mathrm{test}}$. where p, 0<p<1, is a parameter that determines the magnitude of perturbation from the steady state in the allowable range for $b_{i_{\mathrm{test}}}$ [here, p=0.2 is used based on Reznik *et al.* (2013)]. The solutions of problems in Equations (5) and (6) determine the incremental objective function ($Z_{i_{\mathrm{test}}}^{+}$) and decremental objective function ($Z_{i_{\mathrm{test}}}^{-}$), respectively. These calculated values are utilized to determine incremental shadow price ($\lambda_{i_{\mathrm{test}}}^{+}$) and decremental shadow price ($\lambda_{i_{\mathrm{test}}}^{-}$) from Equations (7) and (8) (Reznik *et al.* 2013): where Z is the objective function of FBA [problem in Equation (2)] at steady state. The threshold $\varepsilon =10^{-5}$ is assumed to evaluate:


(5) $$\begin{array}{c} \max\;Z_{i_{\mathrm{test}}}^{+}=c^{T}v,\\ s.t.,\;\mathrm{Sv}=b,\\ v^{\mathrm{LB}}\leq v\leq v^{\mathrm{UB}},\\ b_{i}=\{\begin{array}{cc} p.G_{i_{\mathrm{test}}}^{+}\;\; & ,i=i_{\mathrm{test}}\\ 0\;\; & ,i\neq i_{\mathrm{test}} \end{array}, \end{array}$$

(6) $$\begin{array}{c} \max\;Z_{i_{\mathrm{test}}}^{-}=c^{T}v,\\ s.t.,\;\mathrm{Sv}=b,\\ v^{\mathrm{LB}}\leq v\leq v^{\mathrm{UB}},\\ b_{i}=\{\begin{array}{cc} p.G_{i_{\mathrm{test}}}^{-}\;\; & ,i=i_{\mathrm{test}}\\ 0\;\; & ,i\neq i_{\mathrm{test}} \end{array}, \end{array}$$

(7) $$\lambda_{i_{\mathrm{test}}}^{+}=\frac{Z_{i_{\mathrm{test}}}^{+}-Z}{p.G_{i_{\mathrm{test}}}^{+}},$$

(8) $$\lambda_{i_{\mathrm{test}}}^{-}=\frac{Z_{i_{\mathrm{test}}^{-}}-Z}{p.G_{i_{\mathrm{test}}}^{-}},$$

(9) $$|\lambda_{i_{\mathrm{test}}}^{+}-\lambda_{i_{\mathrm{test}}}^{-}|<\varepsilon .$$

If the calculated shadow price satisfies both conditions in Equations (4) and (10), we consider it as a valid shadow price.

### 2.4 Defining MPI network as the gold standard

Our main goal is to show that the values of shadow prices can lead us to predict MPI. In this regard, we define the MPI network as a gold standard by matrix $I_{m\times n}$, where


(10) $$I_{ik}=\{\begin{array}{cc} 1\; & \text{interaction between}\;M_{i}\in M\;\text{and}\;P_{k}\in P,\\ 0\; & \text{otherwise}. \end{array}$$

The MPI prediction problem has as its input a GEM, and the output consists of predicted $I_{m\times n}^{\prime }$ yielding the MPI network.

### 2.5 Curating GEM

In this study, we execute our method on two organisms and corresponding metabolic models: iJO1366, a genome-scale reconstruction of the metabolic network of *E.coli* str. K-12 substr. MG1655 (Orth *et al.* 2011) and Yeast-GEM, the consensus GEM of *S.cerevisiae* (Lu *et al.* 2019), version 8.5.0 (Sánchez *et al.* 2021). In these models, we performed the following modifications using the COBRA Toolbox (Heirendt *et al.* 2019): (i) we split reversible reactions into two irreversible reactions because both substrates and products may interact with enzymes catalyzing reversible reactions. (ii) iJO1366 model has two biomass reactions, so we remove reaction *BIOMASS_Ec_iJO1366_WT_53p95M* and keep the second biomass reaction *BIOMASS_ Ec_iJO1366_core_53p95M* (Yeast-GEM does not need this step). (iii) We optimize models by considering biomass reactions as objective functions [see problem in Equation (2)], and fix lower bounds of biomass reactions’ flux at 90% of their optimum values. The details of these two models are in Table 1.

**Table 1.** The details of utilized GEMs.^a^

Metabolic model	Organism	Metabolites	Genes	Reaction	Irreversible reactions	Reactions with GPR
iJO1366	*E.coli*	1805	1367	2583	3218	2717
Yeast-GEM	*S.cerevisiae*	2742	1150	4058	5688	3445

a The table presents the number of metabolites, genes, and reactions before and after modifications, along with the number of reactions with GPR rules.

### 2.6 SARTRE framework

In this subsection, we propose a framework named SARTRE to predict MPIs. In brief, we utilize the metabolic model to extract features for both metabolites and proteins. Then, we employ these features to train a random forest (RF) classifier to predict the interaction of unseen metabolite–protein pairs.

**Figure 1.**
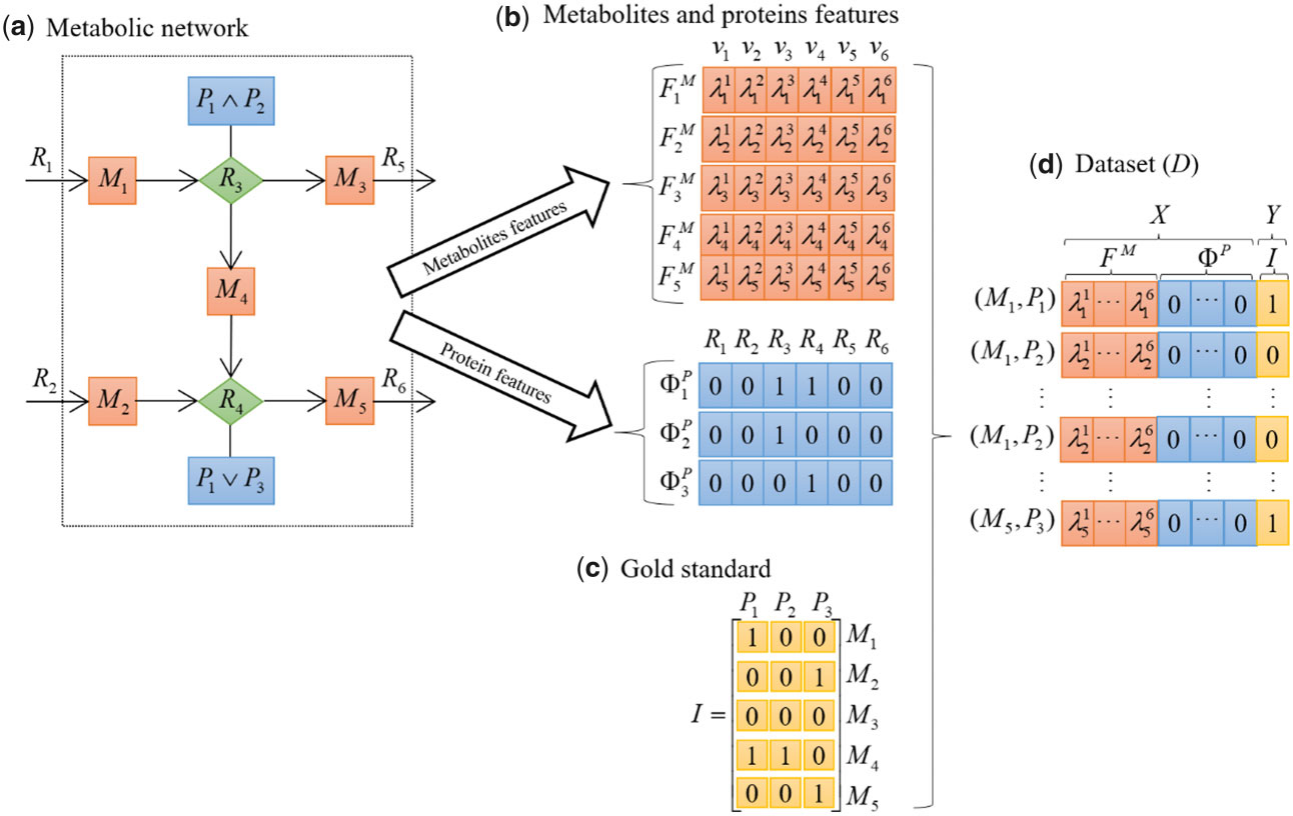
Illustration of the workflow of SARTRE. (a) Toy network of six reactions, five metabolites, and three proteins. Reaction 3 has GPR rule that involves P1 AND P2, while Reaction 3 involves P1 OR P3. (b) Metabolite and protein features are generated based on shadow prices and GPR rules for each reaction, respectively, (c) the gold standard of MPIs is then used with the features to build a classifier (d).

We compute shadow prices of each metabolite as a set of features based on solving the dual problem [problem in Equation (3)]. Here, we show the vector of valid shadow prices for $M_{i}\in M$ as follows: where $\lambda_{i}^{j}$ indicates the calculated shadow price for $M_{i}$ in FBA by maximizing $v_{j}$, where the lower bound of the biomass reaction’s flux is fixed at 90% of its optimum value. We define the dataset D=(X,Y) for classifier RF as follows: where where ⊕ is a concatenation operator, $\Phi_{k}^{P}$ shows protein feature of the protein $P_{k}\in P$, and $I_{ik}$ represents the interaction [see Equation (10)]. The simple example of constructing dataset D is shown in Fig. 1.


(11) $$F_{i}^{M}=[\lambda_{i}^{1},\lambda_{i}^{2},\ldots ,\lambda_{i}^{r}],\;\forall M_{i}\in M,$$

(12) $$(X,Y)=\{(X_{11},Y_{11}),\ldots ,(X_{mn},Y_{mn})\},$$

(13) $$\forall (M_{i},P_{k})\in M\times P,\;X_{ik}=F_{i}^{M}⊕\Phi_{k}^{P},\;Y_{ik}=I_{ik},$$

### 2.7 Data pre-processing

In this part, we process the mentioned databases to make dataset D=(X,Y) as follows:

1. Mapping the names of proteins and metabolites in the GS datasets to the corresponding names of the metabolic models to get the intersection of metabolites and proteins between the metabolic model and GS datasets.
2. Calculating $\lambda_{i}^{j}$ in dual problem [see Equation (3)] for each $R_{j}\in R$ if its GPR includes at least one gene.
3. Evaluating two validation conditions in Equations (4) and (9). If the calculated shadow price is invalid, we replace it with NaN (Not a Number).
4. Selecting each metabolite $M_{i}$, i∈{1,2,…,m}, which includes at least 80% not NaN in the calculated shadow prices. For these metabolites, we replace missing values (NaN) with the original shadow price calculated from problem in Equation (3) and construct $F_{i}^{M}$ [see Equation (11)].
5. Removing redundant protein features ($\Phi^{P}$); some proteins from the dataset may be either in all or in none of the reactions, depending on GPR rules. As a result, redundant features occur, corresponding to columns (reactions) in $\Phi^{P}$.
6. Rounding all shadow prices of $F^{M}$ to two decimal points and taking unique set of features to reduce the feature dimension. The selection of two decimal points balances the trade-off between computation cost and performance. Using more decimal points results in a larger feature size and requires more computation. However, keeping fewer decimal points leads to a worse performance (in terms of accuracy).
7. Taking element-wise average over features of repetitious metabolites and merging them into one entry.
8. Constructing dataset D=(X,Y) by concatenating feature vectors $F^{M}$ and $\Phi^{P}$ [see Equation (1)] to construct X [see Equation (12)] followed by extracting labels of each metabolite–protein pair from GSs to construct Y.

According to four gold standards with corresponding metabolic models, we generate four datasets, $D_{1}$, $D_{2}$, $D_{3}$, and $D_{4}$. The details of these datasets are shown in Table 2.

**Table 2.** Gold standards and metabolic models used by SARTRE for *E.coli* and *S.cerevisiae*.^a^

Dataset	Gold standard	Metabolic model	Metabolites	Proteins	Metabolite–protein pairs
$D_{1}$	Piazza	iJO1366	18	964	17 352
$D_{2}$	Reznik	iJO1366	148	328	48 544
$D_{3}$	STITCH-*E.coli*	iJO1366	29	1365	39 585
$D_{4}$	STITCH-Yeast	Yeast-GEM	41	1150	47 150

a The table details the gold standards and metabolic models used for implementing SARTRE for *E.coli* and *S.cerevisiae*. It includes the number of metabolites, proteins, and metabolite–protein pairs.

### 2.8 RF model

Following the pre-processing step, we have constructed datasets that can be used for downstream classifiers. However, due to the small fraction of MPIs in the gold standards, compared to all possible metabolite–protein pairs in the datasets, we have imbalanced datasets with a greater number of non-interacting metabolite–protein pairs. As a result, the classifier would be biased toward learning the non-interacting class. To avoid this problem, we undersampled the entire dataset 10 times in order to ensure a balanced dataset $D\in \{D_{1},D_{2},D_{3},D_{4}\}$. Consequently, we obtain 10 sample data named $\left\{D_{l}^{1},D_{l}^{2},\ldots ,D_{l}^{10}\right\}$, where l∈{1,2,3,4}. For the evaluation step, we employ 5-fold cross-validation on the undersampled datasets. Finally, we train a RF model with 100 trees on the training set and calculate metrics on the test folds. Finally, we take an average of metrics over 10 undersampled datasets.

## 3 Results and discussion

### 3.1 Formulation of SARTRE

Given a GEM, SARTRE extracts two types of features capturing: (i) protein–reaction associations, based on the GPR rules and (ii) metabolite–reaction flux effects, based on shadow prices. To extract the first type of features, we built a n×r matrix $\Phi^{P}$, with rows corresponding to proteins and columns denoting reactions. The entry $\Phi_{kj}^{P}$ is one whenever protein k is in the GPR rule of reaction j, and zero, otherwise. To derive the features denoting metabolite–reaction flux effects, we rely on shadow prices in the context of FVA (Mahadevan and Schilling 2003). FVA determines the minimum and maximum fluxes that a reaction supports at steady state, given a set of constraints. Due to the prevalence of inhibitory MPIs (Alam *et al.* 2017), we postulate that the shadow price corresponding to steady-state constraint for a metabolite i with respect to maximizing the flux through reaction j is informative of presence/absence of interaction between the metabolite i and (some of) the proteins participating in the GPR rules of reaction j. To further consider physiologically relevant flux distributions, we determine the maximum fluxes under the constraint of ensuring 90% of optimal specific growth rate predicted by FBA. Altogether, we determine each pair of metabolite and protein can then be described by the concatenated vectors of 2r features, gathering the shadow prices and the protein–reaction associations for each reaction. Finally, given a gold standard of MPIs, we can specify the presence/absence of interaction for each pair of considered metabolites and proteins, completing the input for prediction.

For instance, in the toy network on Fig. 1a, consisting of five metabolites of MPIs based on machine-learning classification approaches interconverted by six reactions, catalyzed by altogether three proteins, the corresponding matrices of features for the metabolites and proteins are shown in Fig. 1b. Given a gold standard of interactions (Fig. 1c), the problem is then to use the input dataset of 15 metabolite–protein pairs, along with their labels (0 absence, 1 presence of interaction) to train a classifier (Fig. 1d).

### 3.2 Performance of SARTRE with GEM and gold standards of *E.coli*

To test the performance of SARTRE, we used as input a curated GEM of *E.coli*, iJO1366 (Orth *et al.* 2011), in which all reversible reactions were split into two irreversible reactions. We then determined the shadow prices for each steady-state metabolite constraint with respect to maximization of every flux at 90% of maximum specific growth rate. To test the effect of gold standards, we assembled three datasets for training, obtained from: (i) a recent chemoproteomic approach that systematically recognizes MPIs in their native environment by combining limited proteolysis and mass spectrometry (Piazza *et al.* 2018), (ii) an MPI network (Reznik *et al.* 2017) compiled from BRENDA and BioCyc (Chang *et al.* 2009, 2015), and (iii) the STITCH database of MPIs (Kuhn *et al.* 2008). The corresponding datasets differ with respect to the number of metabolites, proteins, and MPIs they comprise (Table 2). These datasets were pre-processed by consolidating them against the metabolites and proteins included in iJO1366. In building the features, we also ensured that the calculated shadow prices are not degenerate (see Section 2), resulting in the smaller gold standard used for training (Table 2).

While SARTRE allows the usage of any classification approach, in our implementation, we relied on RFs for training the classifier. To balance the two classes (i.e. presence/absence of interaction), we performed 10 random undersamplings. These were then used for training RF classifier with 100 trees on datasets resulting from the undersampling in 5-fold cross-validation. Last, to compare the performance of classifiers with standard features, we used metabolite topological fingerprints of size 128 obtained from RDKit (Landrum 2016) instead of shadow prices.

**Figure 2.**
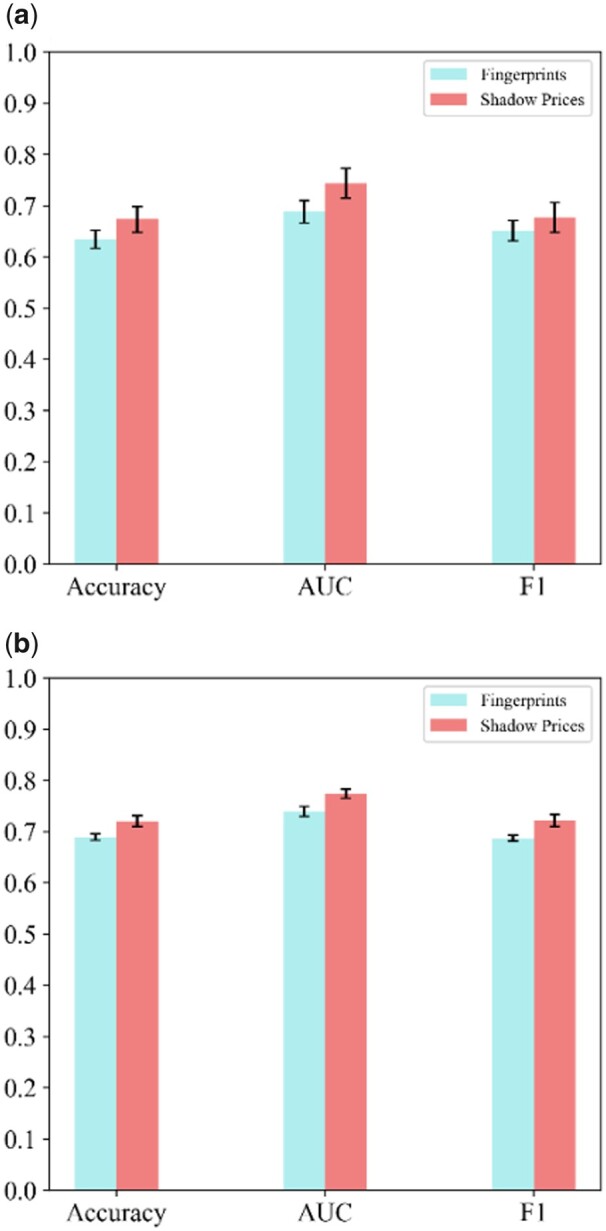
Performance of SARTRE on small gold standards for *E.coli.* The performance of SARTRE with respect to accuracy, AUC, and *F*1-measure is shown for two gold standards for *E.coli*: (a) 765 interacting and 765 randomly chosen metabolite–protein pairs not in the gold standard (assumed to be non-interacting), with 800 protein features, 166 shadow price features, and fingerprints of size 128 for the metabolites ($D_{1}$ in Table 3) and (b) 993 interacting and non-interacting metabolite–protein pairs, with 333 protein features, 320 shadow price features, and 128-digit fingerprints for the metabolites ($D_{2}$ in Table 3).

Following this approach with the gold standard from Piazza *et al.* (2018), we found that SARTRE significantly outperformed the classifier based on fingerprints with respect to all three measures of performance (*P*-value <1e-5), namely accuracy, area under the receiver operating curve (AUC), and *F*1-measure (Fig. 2a). Analogous findings were obtained when using the database of MPIs compiled from BRENDA and BioCyc (Fig. 2b), with *P*-value <1e-7, which demonstrates the added value of considering shadow prices as an important feature in predicting MPIs. Further, with the gold standard from STITCH, we also investigated the effect of confidence for the MPIs on the predictions. To this end, we extracted MPIs with four different levels of confidence, namely: low, medium, high, and highest. With the four gold standards of different confidence obtained from STITCH, we applied SARTRE and again compared the effect of using shadow prices. In these cases, the performance of SARTRE was comparable to that of using fingerprints as features (Table 4 and Supplementary Table S1).

**Table 4.** Performance of SARTRE on gold standards from STITCH.^a^

Dataset	Confidence score	Pairs per class	Metabolite feature	$\left|F^{M}\right|$	$\left|\Phi^{P}\right|$	Accuracy	AUC	*F*1-measure
$D_{3}$ (*E.coli*)	400	3996	Shadow price	209	1000	0.825 ± 0.003	0.889 ± 0.003	0.835 ± 0.002
			Fingerprint	128	1000	0.816 ± 0.004	0.874 ± 0.004	0.826 ± 0.003
	700	2319	Shadow price	209	1000	0.815 ± 0.006	0.881 ± 0.004	0.826 ± 0.006
			Fingerprint	128	1000	0.802 ± 0.006	0.855 ± 0.006	0.815 ± 0.005
$D_{4}$ (*S.cerevisiae*)	400	4065	Shadow price	265	864	0.835 ± 0.002	0.894 ± 0.002	0.835 ± 0.002
			Fingerprint	128	864	0.835 ± 0.003	0.890 ± 0.003	0.834 ± 0.002
	700	1879	Shadow price	265	864	0.801 ± 0.006	0.865 ± 0.004	0.803 ± 0.006
			Fingerprint	128	864	0.800 ± 0.005	0.863 ± 0.005	0.803 ± 0.005

a The performance of SARTRE with respect to accuracy, AUC, and *F*1-measure is shown for STITCH gold standards for *E.coli* with 3996 metabolite–protein interacting, with medium confidence score (0.4), and 3996 randomly chosen metabolite–protein pairs not in the gold standard (assumed to be non-interacting), and 2319 metabolite–protein interacting and the same number of randomly selected non-interacting metabolite–protein pairs with high confidence score (0.7); 1000 protein features, 209 shadow prices and fingerprints of size 128 for metabolites. The table also includes the performance on the STITCH gold standard for *S.cerevisiae* with 4065 metabolite–protein interacting and the same number of randomly selected (non-interacting) metabolite–protein pairs with medium confidence score (0.4) and 1879 metabolite–protein interacting and the same number of randomly selected (non-interacting) metabolite–protein pairs with high confidence score (0.7); 864 protein features, 265 shadow prices and fingerprint for size 128 for the metabolites.

Moreover, label permutation and feature permutation are executed on three *E.coli* datasets to construct null distributions (Ojala and Garriga 2010), which are taken as the input of the classifier. Results in Supplementary Table S9 demonstrated that the classifier performs at random and the null hypothesis is rejected with significant *P*-values (*P*-value < 10e^−7^). To conclude, extracted features of metabolites and proteins are valuable for predicting MPIs. Moreover, the RF classifier learns strong connections between data and labels, and dependency of features.

### 3.3 Performance of SARTRE with GEM and gold standards of *S.cerevisiae*

Next, we assessed SARTRE with gold standard and metabolic network for *S.cerevisiae*. To this end, we utilized the curated metabolic model Yeast-GEM (Lu *et al.* 2019) in which all reversible reactions were split into two irreversible reactions, and the biomass reaction was fixed on 90% of its maximum flux. We employed STITCH database of MPIs of *S.cerevisiae* as our gold standard. We then trained a RF classifier on the dataset; due to imbalance labels, we performed 10 random undersamplings. On each of them, we trained and evaluated SARTRE with 5-fold cross-validation. Finally, we compared the results with the same workflow, except that fingerprints were utilized as metabolite features. Like in the analysis of *E.coli* datasets, we used accuracy, AUC, and *F*1-measure to compare the results of SARTRE with the fingerprint as metabolite features. The results showed that using shadow prices or fingerprints led to comparable performance in comparison to approaches based on fingerprints only based on the different measures used (Table 4). Similar permutation tests of Section 3.2 are executed on yeast dataset and results are available in Supplementary Table S9.

### 3.4 Performance of the specifies-specific classifiers on shared MPIs

To showcase the added value of using shadow prices to predict MPIs, we performed two additional analyses to assess if shadow prices obtained from different metabolic networks affect the prediction of same MPIs and if shadow prices from the same network affect the prediction of MPIs in different metabolic subsystems.

For the first analysis, we compared the predictions for the same set of metabolite–protein pairs on RF models trained with features obtained from the metabolic models of *E.coli* and *S.cerevisiae*, i.e. iJO1366 and Yeast-GEM, respectively. To this end, we first used four metabolites (namely, three inorganic molecules: sulfate, chloride, and magnesium, as well as D-glucose), and considered 282 proteins whose orthologues are present in both models. We then used the gold standards of the two organisms from STITCH with medium confidence score (STITCH-*E.coli* and STITCH-Yeast, respectively). In total, we extracted all 1128 labels (i.e. interacting or non-interacting) of the metabolite–protein pairs with medium confidence score from the gold standards, of which 955 pairs had the same label in the two gold standards. We kept these 955 pairs to create a test set, and excluded the remaining 173 pairs from the test set to allow comparability of the predictions based on the features from the two different models. We then trained two separate models with 7086 and 7224 metabolite–protein pairs in *E.coli* and *S.cerevisiae*, respectively, and predicted MPIs on the test set. We found that the accuracies of predictions are 0.754 and 0.755 for *E.coli* and *S.cerevisiae*, similar to what was observed in the previous case studied. To compare the predictions of two separate models, we calculated the cosine similarity of two prediction vectors. Cosine similarity, which measures the similarity between two vectors by using the cosine of the angle between them, is defined for two vectors $V_{1}$ and $V_{2}$ as below:


(14) $$\text{Cosine Similarity}(V_{1},V_{2})=\text{cos}\;\theta =\frac{V_{1}.V_{2}}{\mathrm{||}V_{1}||\;||V_{2}||}.$$

We found that the cosine similarity between predictions of two models is 0.980, demonstrating that the shared MPIs can be learned from two different organisms with features extracted from their respective metabolic models.

In the second analyses, we investigated the predictions of MPIs for metabolite–protein pairs in two metabolic subsystems of the iJO1366 model, namely, “Alternate Carbon Metabolism” and “Cofactor and Prosthetic Group Biosynthesis,” for which we found a larger number of MPIs in the gold standard in comparison to the other pathways. For each of the mentioned subsystems, we determined the intersection between the STITCH gold standard for *E.coli* with medium confidence score and the metabolites in each of these subsystems. We used the resulting metabolite–protein pairs as a test set, and to generate a training set we performed random undersampling on the remaining pairs from the gold standard (for the total number of instances, see Supplementary Table S2). We found that the accuracy dropped to 0.615 and 0.815, due to the larger number of false positives. Importantly, this analysis indicated that shadow prices covering different metabolic systems increase the accuracy of predictions.

### 3.5 Comparison with existing MPI predictions

In the following, we compared the performance of SARTRE with that of the deep-learning approach used in Zhao *et al.* (2021), whose ∼50 000 PMP predictions for *E.coli* and *S.cerevisiae* (and two other species, human and mouse) are based on features, obtained from a protein–protein interaction network, for 9631 proteins as well as different representations and fingerprints for 23 metabolites. The comparison is based on the four gold standards (Table 3) using three performance metrics, namely accuracy, (macro) AUC, and (macro) *F*1 (Supplementary Table S3). Our comparative analyses demonstrated that SARTRE outperformed the deep-learning approaches with respect to macro AUC in all gold standards (with exceptions to the stringent STITCH-*E.coli* with a cut-off of 150 and 700) (Table 5). The macro *F*1 of SARTRE was comparable of larger than that of the deep-learning approach in all but one comparison, further demonstrating the added value of the proposed approach that couples machine learning with constraint-based modeling to predict MPIs. For fairness of comparison, we made sure that in both cases the test sets remained untouched and as a result unbalanced. Accordingly, the performance of SARTRE, presented in Table 5, is slightly lower than the case where balanced test sets were used (Table 4).

**Table 3.** Properties of the gold standards of MPIs.^a^

Gold standard	Organism	Metabolites	Proteins	Interactions
Piazza	*E.coli*	20	2559	1678
Reznik	*E.coli*	321	364	1669
STITCH-*E.coli*	*E.coli*	88 044	4028	2 278 769
STITCH-Yeast	*S.cerevisiae*	177 977	5845	3 533 097

a The table contains the number of metabolites, proteins, and MPIs in four gold standards for *E.coli* and *S.cerevisiae*.

**Table 5.** Comparison of metrics with MPI predictions from a deep-learning approach.^a^

Dataset	Confidence score	Zhao *et al.*	SARTRE
Piazza		0.61	0.68
Reznik		0.54	0.73
STITCH-*E.coli*	150	0.77	0.77
	400	0.82	0.82
	700	0.84	0.82
	900	0.75	0.77
STITCH-Yeast	150	0.63	0.76
	400	0.48	0.84
	700	0.7	0.76
	900	0.58	0.75

a The performance of SARTRE on four constructed datasets is compared to previous MPI predictions from Zhao *et al.* (2021) that relies on a deep-learning model with an extensive set of features. Zhao *et al.* use metabolite features with the size of 2325 for all datasets, and protein features with the size of 964, 328, 1365, and 1150, respectively for the four datasets. On the other hand, SARTRE uses metabolite features with the size of 168, 320, 209, and 265, and protein features with the size of 800, 333, 1000, and 864, respectively, for the four datasets. Macro AUC is calculated based on the predictions on test sets, using 10-fold cross-validation.

### 3.6 Performance of SARTRE in different media compositions

Since shadow prices may change with media composition, here, we assess the sensitivity of SARTRE as media composition changes. To this end, we examined SARTRE with two different changes of media composition, namely by changing the carbon sources and the limitation of critical nutrients.

First, we applied SARTRE with different carbon sources of the IJO1366 model and employ STITCH with the medium confidence score of 400. More specifically, our primary results were based on the glucose carbon source, while here we examine other carbon sources namely, acetate, fructose, glycerol, mannose, and succinate. Based on the media compositions, different metabolites would be retained due to the pre-processing approach and as a result, we have different sizes of metabolite features and number of metabolite–protein pairs per class. As shown in Supplementary Table S4, SARTRE performance is not influenced by different media compositions.

Furthermore, we examined the MPI predictions in different media compositions to identify the robustness of MPIs to different compositions, by distinguishing predicted MPIs across all compositions and pairs that are specific to only one of them. To this end, each MPI is given a score between 0 and 10, which reflects the number of times that this pair is predicted as positive in 10-folds of each undersampled dataset. Eventually, for the comparison between different compositions, we extract interacting pairs that are always predicted as positive and therefore receive a score of 10. By implementing this approach, we found that 2819 positive pairs were predicted in all examined media compositions. However, some MPIs are predicted only in one condition and are specific to that media composition (see Supplementary Table S4 for more details).

Second, to assess the sensitivity of SARTRE to the limitation of critical nutrients, we considered three key nutrients, namely carbon, nitrogen, and phosphorus. Consequently, we selected the following exchange reactions out of the growth-supporting reactions; for carbon source: D-glucose, glycerol, and sucrose, for nitrogen source: ammonia, L-arginine, and L-glutamine, for phosphorus source: phosphate and phosphonate.

It is important to note that for each of the mentioned sources, only one growth-supporting reaction has a non-zero uptake rate at a given time. Consequently, the biomass flux rate is decreased by limiting the uptake of each of the key nutrients (carbon/nitrogen/phosphorus), which can be achieved by constraining its rate of the corresponding uptake reaction.

To better specify the effect of the restriction on the uptake rates to the value of the biomass flux, we define limiting and non-limiting uptake rates. If the uptake rate of the source results in a specific growth rate (i.e. flux through the biomass reaction) smaller than 0.9 of the optimum, predicted by the model, we consider the uptake value as limiting to growth; otherwise, the uptake value is considered non-limiting.

Next, we examined the performance of SARTRE for each of the mentioned growth-supporting reactions with three limiting uptake rates and four non-limiting ones, according to the definitions above.

We consider the default iJO1366 as our baseline GEM, in which there is only one growth-supporting reaction (i.e. glucose, ammonia, and phosphate) with non-zero uptake rate for these three key nutrients. In the baseline, the optimum flux through the biomass reaction equals 0.9824 mmol gDW^−1^ h^−1^, and, based on the definition above, we take the threshold of 0.8842 mmol gDW^−1^ h^−1^ to differentiate uptake rates that are limiting and non-limiting to growth. Supplementary Tables S5–S7 demonstrate that SARTRE performance is not sensitive to the limitations of important nutrients.

### 3.7 Gold-standard MPIs that are trivially present in the GEMs

In this section, for the four gold standards used in our study, we investigate pairs that can be also obtained directly from the GEMs. First, to obtain the metabolite–protein relations from the GEMs, for each reaction, we assume that there is an interaction between each substrate/product and genes encoding enzymes in the GPR rule. These sets of interactions are then compared to the ones from the gold standards (see Supplementary Table S8). Coverage between MPIs that are obtained directly from the GEMs and those of gold standards varies between 4.35% of STITCH-*E.coli* with a low confidence score, and 41.79% of STITCH-yeast with the highest confidence score. To ensure the consistency of SARTRE, we excluded such trivial MPIs from the gold-standard datasets and re-evaluated the performance of SARTRE. In the minimum coverage case, metrics changed from 0.76, 0.83, and 0.77 to 0.76, 0.83, and 0.77 for accuracy, AUC, and *F*1-measure, respectively. In the maximum coverage case, the metrics changed from 0.75, 0.84, and 0.76 to 0.75, 0.83, and 0.76 for accuracy, AUC, and *F*1-measure, respectively. The same approach is applied to the other gold-standard datasets and their results are available in Supplementary Table S8. Results demonstrated that SARTRE is not sensitive to the shared MPIs between GEM and gold standards.

## 4 Conclusion

Despite recent research efforts, understanding the functional role of MPIs in modulating different cellular processes remains challenging. Machine and deep-learning approaches have provided advances in prediction of MPIs based on structural and ontology-based features. Our study adds to these advances by predicting MPIs in the context of metabolic networks and effects they have on metabolic fluxes. To this end, we expanded the usage of shadow prices in an innovative way to predict MPIs in metabolic networks using machine-learning approaches. We demonstrated that SARTRE results in an improvement of prediction performance in comparison to the usage of metabolic fingerprints and shows that shadow prices are the features that contribute most to the predictions. In addition, our comparative analyses showed that SARTRE is competitive against a recent study that used deep learning with a variety of features to predict MPIs. In the future, SARTRE can be extended to consider ensemble of weak classifiers shown to improve prediction performance in many applications. Due to the usage of constraint-based modeling formulation for the extracted features, SARTRE paves the way for improving the understanding of MPIs by further developments in this modeling framework and its application across other species for which metabolic network models of high quality have already been assembled and analyzed.

## Supplementary Material

vbad098_Supplementary_Data

## Data Availability

The data underlying this article are available in GitHub at https://github.com/fayazsoleymani/SARTRE.

## References

[vbad098-B1] Akbari A , Yurkovich JT, Zielinski DC et al The quantitative metabolome is shaped by abiotic constraints. Nat Commun 2021;12:3178. 10.1038/s41467-021-23214-9. 34039963 PMC8155068

[vbad098-B2] Alam MT , Olin-Sandoval V, Stincone A et al The self-inhibitory nature of metabolic networks and its alleviation through compartmentalization. Nat Commun 2017;8:16018. 10.1038/ncomms16018. 28691704 PMC5508129

[vbad098-B3] Bordbar A , Monk JM, King ZA et al Constraint-based models predict metabolic and associated cellular functions. Nat Rev Genet 2014;15:107–20. 10.1038/nrg3643. 24430943

[vbad098-B4] Chang A , Scheer M, Grote A et al BRENDA, AMENDA and FRENDA the enzyme information system: new content and tools in 2009. Nucleic Acids Res 2009;37:D588–92. 10.1093/nar/gkn820. 18984617 PMC2686525

[vbad098-B5] Chang A , Schomburg I, Placzek S et al BRENDA in 2015: exciting developments in its 25th year of existence. Nucleic Acids Res 2015;43:D439–46. 10.1093/nar/gku1068. 25378310 PMC4383907

[vbad098-B6] Diether M , Nikolaev Y, Allain FH et al Systematic mapping of protein‐metabolite interactions in central metabolism of *Escherichia coli*. Mol Syst Biol 2019;15:1–16. 10.15252/msb.20199008. PMC6706640 31464375

[vbad098-B7] Diether M , Sauer U. Towards detecting regulatory protein–metabolite interactions. Curr Opin Microbiol 2017;39:16–23. 10.1016/j.mib.2017.07.006. 28810194

[vbad098-B8] Hackett SR , Zanotelli VRT, Xu W et al Systems-level analysis of mechanisms regulating yeast metabolic flux. Science 2016;354:aaf2786. 10.1126/science.aaf2786. 27789812 PMC5414049

[vbad098-B9] Heirendt L , Arreckx S, Pfau T et al Creation and analysis of biochemical constraint-based models using the COBRA toolbox v. 3.0. Nat Protoc 2019;14:639–702. 30787451 10.1038/s41596-018-0098-2 PMC6635304

[vbad098-B10] Henry CS , Broadbelt LJ, Hatzimanikatis V. Thermodynamics-based metabolic flux analysis. Biophys J 2007;92:1792–805. 10.1529/biophysj.106.093138. 17172310 PMC1796839

[vbad098-B11] Kuhn M , von Mering C, Campillos M et al STITCH: interaction networks of chemicals and proteins. Nucleic Acids Res 2008;36:D684–8. 10.1093/nar/gkm795. 18084021 PMC2238848

[vbad098-B12] Li X , Gianoulis TA, Yip KY et al Extensive in vivo metabolite-protein interactions revealed by large-scale systematic analyses. Cell 2010;143:639–50. 10.1016/j.cell.2010.09.048. 21035178 PMC3005334

[vbad098-B13] Li X , Snyder M. Metabolites as global regulators: a new view of protein regulation: systematic investigation of metabolite-protein interactions may help bridge the gap between genome-wide association studies and small molecule screening studies. Bioessays 2011;33:485–9. 10.1002/bies.201100026. 21495048

[vbad098-B14] Link H , Kochanowski K, Sauer U. Systematic identification of allosteric protein-metabolite interactions that control enzyme activity in vivo. Nat Biotechnol 2013;31:357–61. 10.1038/nbt.2489. 23455438

[vbad098-B15] Lu H , Li F, Sánchez BJ et al A consensus *S. cerevisiae* metabolic model Yeast8 and its ecosystem for comprehensively probing cellular metabolism. Nat Commun 2019;10:3586. 10.1038/s41467-019-11581-3. 31395883 PMC6687777

[vbad098-B16] Luzarowski M , Vicente R, Kiselev A et al Global mapping of protein–metabolite interactions in *Saccharomyces cerevisiae* reveals that Ser-Leu dipeptide regulates phosphoglycerate kinase activity. Commun Biol 2021;4:181. 33568709 10.1038/s42003-021-01684-3 PMC7876005

[vbad098-B17] Mahadevan R , Schilling CH. The effects of alternate optimal solutions in constraint-based genome-scale metabolic models. Metab Eng 2003;5:264–76. 10.1016/j.ymben.2003.09.002. 14642354

[vbad098-B18] Maranas CD , Zomorrodi AR. Flux balance analysis and LP problems. In: Optimization Methods in Metabolic Networks. John Wiley & Sons, Inc., 2016, 53–80. 10.1002/9781119188902.ch3.

[vbad098-B19] Ojala M , Garriga GC. Permutation tests for studying classifier performance. J Mach Learn Res 2010;11:1833–63.

[vbad098-B20] Orsak T , Smith TL, Eckert D et al Revealing the allosterome: systematic identification of metabolite-protein interactions. Biochemistry 2012;51:225–32. 10.1021/bi201313s. 22122470

[vbad098-B21] Orth JD , Conrad TM, Na J et al A comprehensive genome-scale reconstruction of *Escherichia coli* metabolism-2011. Mol Syst Biol 2011;7:1–9. 10.1038/msb.2011.65. PMC3261703 21988831

[vbad098-B22] Orth JD , Thiele I, Palsson BO. What is flux balance analysis? Nat Biotechnol 2010;28:245–8. 10.1038/nbt.1614. 20212490 PMC3108565

[vbad098-B23] Palsson BO , Yurkovich JT. Is the kinetome conserved? Mol Syst Biol 2022;18:e10782. 10.15252/msb.202110782. 35188334 PMC8859747

[vbad098-B24] Piazza I , Kochanowski K, Cappelletti V et al A map of protein-metabolite interactions reveals principles of chemical communication. Cell 2018;172:358–72.e23. 10.1016/j.cell.2017.12.006. 29307493

[vbad098-B25] Razaghi-Moghadam Z , Sokolowska EM, Sowa MA et al Combination of network and molecule structure accurately predicts competitive inhibitory interactions. Comput Struct Biotechnol J 2021;19:2170–8. 10.1016/j.csbj.2021.04.012. 34136091 PMC8172118

[vbad098-B26] Landrum, G. RDKit: Open-Source Cheminformatics Software. 2016.

[vbad098-B27] Reznik E , Christodoulou D, Goldford JE et al Genome-scale architecture of small molecule regulatory networks and the fundamental Trade-Off between regulation and enzymatic activity. Cell Rep 2017;20:2666–77. 10.1016/j.celrep.2017.08.066. 28903046 PMC5600504

[vbad098-B28] Reznik E , Mehta P, Segrè D. Flux imbalance analysis and the sensitivity of cellular growth to changes in metabolite pools. PLoS Comput Biol 2013;9:e1003195. 10.1371/journal.pcbi.1003195. 24009492 PMC3757068

[vbad098-B29] Sánchez B , Kerkhoven E, Anton M et al; feiranl eiden309. SysBioChalmers/yeast-GEM: yeast 8.5.0. 10.5281/zenodo.5062615. 2021.

[vbad098-B30] Scheer M , Grote A, Chang A et al BRENDA, the enzyme information system in 2011. Nucleic Acids Res 2011;39:D670–6. 10.1093/nar/gkq1089. 21062828 PMC3013686

[vbad098-B31] Varma A , Palsson BO. Stoichiometric flux balance models quantitatively predict growth and metabolic by-product secretion in wild-type *Escherichia coli* W3110. Appl Environ Microbiol 1994;60:3724–31. 10.1128/aem.60.10.3724-3731.1994. 7986045 PMC201879

[vbad098-B32] Winston WL , Goldberg JB. Operations Research: Applications and Algorithms. Vol. 3. Thomson Brooks/Cole Belmont, 2004.

[vbad098-B33] Zhao L , Zhu Y, Wang J et al A brief review of protein-ligand interaction prediction. Comput Struct Biotechnol J 2022;20:2831–8. 35765652 10.1016/j.csbj.2022.06.004 PMC9189993

[vbad098-B34] Zhao T , Liu J, Zeng X et al Prediction and collection of protein-metabolite interactions. Brief Bioinform 2021;22:bbab014. 10.1093/bib/bbab014. 33554247

