# Supplementary material for: Prediction of metabolite–protein interactions based on integration of machine learning and constraint-based modeling
Source: Bioinform Adv. 2023 Jul 17;3(1):vbad098. doi: 10.1093/bioadv/vbad098 (PMC10374491; doi:10.1093/bioadv/vbad098)
Supplement: vbad098_Supplementary_Data [file vbad098_supplementary_data.docx]

# Supplementary Data

**Table S1. Performance of SARTRE on gold standards from STITCH.** The performance of SARTRE with respect to accuracy, AUC, and F1-measure is shown for STITCH gold standards for *E. coli* with 7417 metabolite-protein interacting and the same number of randomly selected non-interacting metabolite-protein pairs with low confidence score (0.15), and 886 metabolite-protein interacting and the same number of randomly selected non-interacting metabolite-protein pairs with highest confidence score (0.9); 1000 protein features, 209 shadow prices and fingerprint of size 128 for the metabolites. The table also includes the performance based on the STITCH gold standard for *S. cerevisiae* with 7348 metabolite-protein interacting and the same number of randomly chosen non-interacting metabolite-protein pairs with low confidence score (0.15) and 907 metabolite-protein interacting and 907 randomly selected non-interacting metabolite-protein pairs with highest confidence score (0.9); 864 protein features, 265 shadow prices and fingerprint of size 128 for metabolites.

| Dataset | Confidence score | Pairs per class | Metabolite feature | $\vert F^{M}\vert$ | $\vert\Phi^{P}\vert$ | Accuracy | AUC | F1-measure |
| --- | --- | --- | --- | --- | --- | --- | --- | --- |
| $D_{3}$ (*E. coli*) | 150 | 7,414 | shadow price | 209 | 1000 | 0.763 | 0.827 | 0.774 |
|  |  |  | fingerprint | 128 | 1000 | 0.763 | 0.832 | 0.766 |
|  | 900 | 866 | shadow price | 209 | 1000 | 0.767 | 0.861 | 0.780 |
|  |  |  | fingerprint | 128 | 1000 | 0.760 | 0.838 | 0.781 |
| $D_{4}$ (S. cerevisiae) | 150 | 7,348 | shadow price | 265 | 864 | 0.755 | 0.828 | 0.743 |
|  |  |  | fingerprint | 128 | 864 | 0.748 | 0.822 | 0.740 |
|  | 900 | 907 | shadow price | 265 | 864 | 0.747 | 0.829 | 0.756 |
|  |  |  | fingerprint | 128 | 864 | 0.753 | 0.835 | 0.762 |

**Table S2. Performance of specific-specific classifiers on shared MPIs.** The performance of SARTRE on two subsystems Alternate Carbon Metabolism and Cofactor and Prosthetic Group Biosynthesis, which are in metabolic model iJO1366 of E. coli. The table includes details of mentioned subsystems as number of metabolites and genes, number of entries in test and train set and accuracy of trained models on test sets.

| Subsystem | Metabolites | Genes | Test set | Train set | Accuracy |
| --- | --- | --- | --- | --- | --- |
| Alternate Carbon Metabolism | 8 | 178 | 1,424 | 7,504 | 0.615 |
| Cofactor and Prosthetic Group Biosynthesis | 15 | 179 | 2,685 | 7,526 | 0.815 |

**Table S3. Comparison of metrics with existing MPI predictions.** The performance of SARTRE on four constructed datasets is compared to previous MPI predictions from Zhao et al. Accuracy, macro AUC, and macro F1-measure are calculated based on the predictions on test sets, using 10-fold cross validation. Zhao et al uses metabolite features with the size of 2325 for all datasets, and protein features with the size of 964, 328, 1365, and 1150, respectively for the four datasets. On the other hand, SARTRE uses metabolite features with the size of 168, 320, 209, and 265, and protein features with the size of 800, 333, 1000, and 864, respectively for the four datasets.

| Dataset | Confidence score | Zhao et al. | | | SARTRE | | |
| --- | --- | --- | --- | --- | --- | --- | --- |
|  |  | Accuracy | macro AUC | macro-F1 | Accuracy | macro AUC | macro F1 |
| Piazza | - | 0.956 | 0.608 | 0.488 | 0.690 | 0.676 | 0.484 |
| Reznik | - | 0.980 | 0.537 | 0.495 | 0.718 | 0.732 | 0.465 |
| STITCH-*E. coli* | 150 | 0.812 | 0.771 | 0.563 | 0.737 | 0.765 | 0.679 |
|  | 400 | 0.891 | 0.821 | 0.540 | 0.776 | 0.824 | 0.652 |
|  | 700 | 0.935 | 0.836 | 0.483 | 0.751 | 0.817 | 0.572 |
|  | 900 | 0.982 | 0.747 | 0.495 | 0.715 | 0.771 | 0.472 |
| STITCH-Yeast | 150 | 0.846 | 0.633 | 0.478 | 0.781 | 0.756 | 0.683 |
|  | 400 | 0.911 | 0.479 | 0.477 | 0.847 | 0.840 | 0.697 |
|  | 700 | 0.958 | 0.694 | 0.489 | 0.781 | 0.756 | 0.683 |
|  | 900 | 0.985 | 0.577 | 0.496 | 0.697 | 0.749 | 0.455 |

**Table S4. Assessing SARTRE on different media compositions of IJO1366, using STITCH *E. coli* medium confidence score.** Details of performing SARTRE on different media compositions including the number of metabolites and their feature size, number of metabolite-protein pairs in each class after random undersampling, and evaluating metrics, which are accuracy, AUC, and F1-measure. The number of proteins in all situations is equal to 1365, whose feature size is equal to 1000. In the next columns, the number of pairs that are predicted at least one time out of ten is shown, followed by pairs that are predicted in every undersampled dataset. The last column reveals pairs that are predicted completely in all undersampled datasets, and are specific predicted MPIs of the carbon source.

| Carbon Source | Metabolites | $\mathbf{\vert F}^{\mathbf{M}}\mathbf{\vert}$ | Pairs per class | Accuracy | AUC | F1-measure | Predicted | Completely predicted | | Specific MIPs |
| --- | --- | --- | --- | --- | --- | --- | --- | --- | --- | --- |
| Glucose | 29 | 209 | 3996 | 0.825 | 0.889 | 0.835 | 3806 | | 3120 | 37 |
| Acetate | 25 | 200 | 3903 | 0.813 | 0.879 | 0.826 | 3623 | | 3000 | 11 |
| Fructose | 29 | 211 | 3964 | 0.827 | 0.888 | 0.837 | 3799 | | 3101 | 24 |
| Glycerol | 30 | 194 | 4636 | 0.827 | 0.889 | 0.838 | 4300 | | 3681 | 620 |
| Mannose | 29 | 212 | 3973 | 0.829 | 0.888 | 0.836 | 3775 | | 3114 | 37 |
| Succinate | 28 | 200 | 3942 | 0.822 | 0.886 | 0.833 | 3707 | | 3088 | 24 |

**Table S5. Evaluation of SARTRE on different carbon sources with uptake rates non-limiting and limiting to growth.** Results are obtained by selecting one carbon source at a time from sources that are supporting growth. D-glucose is non-limiting between ranges of 9.0131 to 10 mmol gDW^-1^h^-1^ and it becomes limiting with uptake rates below 9.0131 mmol gDW^-1^h^-1^. Glycerol is non-limiting with uptake rate between 15.5766 and 17.2814 mmol gDW^-1^h^-1^ and becomes limiting with uptake rates below 15.5733 mmol gDW^-1^h^-1^. Sucrose is non-limiting with uptake rates between 4.5066 to 5 mmol gDW^-1^h^-1^ and becomes limiting with uptake rates lower than 4.5066 mmol gDW^-1^h^-1^. The number of proteins in all situations is equal to 1365, whose feature size is equal to 1000.

| reaction | reaction name | uptake rate  ($\frac{mmol}{gDW\cdot h})$ | limiting/  non-limiting | biomass flux ($\frac{mmol}{gDW\cdot h})$ | metabolites | $\mathbf{\vert F}^{\mathbf{M}}\mathbf{\vert}$ | Pairs per class | ACC | AUC | F1-measure |
| --- | --- | --- | --- | --- | --- | --- | --- | --- | --- | --- |
| EX_glc__D_e | D-Glucose exchange | 2.5 | limiting | 0.2358 | 27 | 203 | 3981 | 0.814 | 0.879 | 0.825 |
|  |  | 5.0 |  | 0.4847 | 29 | 208 | 3996 | 0.823 | 0.886 | 0.834 |
|  |  | 7.5 |  | 0.7335 | 29 | 208 | 3996 | 0.826 | 0.885 | 0.832 |
|  |  | 9.25 | non-limiting | 0.9077 | 29 | 209 | 3996 | 0.823 | 0.887 | 0.833 |
|  |  | 9.5 |  | 0.9326 | 29 | 209 | 3996 | 0.823 | 0.888 | 0.834 |
|  |  | 9.75 |  | 0.9575 | 29 | 209 | 3996 | 0.822 | 0.886 | 0.832 |
|  |  | 10 (baseline) |  | 0.9824 | 29 | 209 | 3996 | 0.825 | 0.889 | 0.835 |
| EX_glyc_e | Glycerol exchange | 4.0 | limiting | 0.2170 | 28 | 188 | 4621 | 0.820 | 0.883 | 0.831 |
|  |  | 8.0 |  | 0.4475 | 30 | 194 | 4636 | 0.828 | 0.889 | 0.838 |
|  |  | 12.0 |  | 0.6780 | 30 | 194 | 4636 | 0.828 | 0.891 | 0.838 |
|  |  | 16.0 | non-limiting | 0.9085 | 30 | 194 | 4636 | 0.828 | 0.891 | 0.839 |
|  |  | 16.4 |  | 0.9316 | 30 | 194 | 4636 | 0.826 | 0.888 | 0.836 |
|  |  | 16.8 |  | 0.9546 | 30 | 194 | 4636 | 0.828 | 0.889 | 0.839 |
|  |  | 17.2814 |  | 0.9824 | 30 | 194 | 4636 | 0.827 | 0.889 | 0.838 |
| EX_sucr_e | Sucrose exchange | 1.25 | limiting | 0.2358 | 30 | 201 | 4049 | 0.824 | 0.887 | 0.834 |
|  |  | 2.5 |  | 0.4847 | 31 | 204 | 4058 | 0.826 | 0.888 | 0.837 |
|  |  | 3.75 |  | 0.7335 | 31 | 204 | 4058 | 0.826 | 0.889 | 0.837 |
|  |  | 4.625 | non-limiting | 0.9077 | 31 | 205 | 4058 | 0.825 | 0.888 | 0.834 |
|  |  | 4.75 |  | 0.9326 | 31 | 205 | 4058 | 0.827 | 0.890 | 0.837 |
|  |  | 4.875 |  | 0.9575 | 31 | 205 | 4058 | 0.828 | 0.891 | 0.837 |
|  |  | 5.0 |  | 0.9824 | 31 | 205 | 4058 | 0.825 | 0.889 | 0.835 |

**Table S6. Evaluation of SARTRE on different nitrogen sources with uptake rates non-limiting and limiting to growth.** Results are obtained by selecting one nitrogen source at a time from sources that are supporting growth. Ammonia is non-limiting between ranges of 9.5494 to 10.6104 mmol gDW^-1^h^-1^ and it becomes limiting with uptake rates below 9.5494 mmol gDW^-1^h^-1^. L-arginine is non-limiting with uptake rate between 2.3873 and 2.6526 mmol gDW^-1^h^-1^ and becomes limiting with uptake rates below 2.3873 mmol gDW^-1^h^-1^. L-Glutamine is non-limiting with uptake rates between 4.7747 to 5.3052 mmol gDW^-1^h^-1^ and becomes limiting with uptake rates lower than 4.7747 mmol gDW^-1^h^-1^. The number of proteins in all situations is equal to 1365, whose feature size is equal to 1000.

| reaction | reaction name | uptake rate  ($\frac{mmol}{gDW\cdot h})$ | limiting/  non-limiting | biomass flux ($\frac{mmol}{gDW\cdot h})$ | metabolites | $\mathbf{\vert F}^{\mathbf{M}}\mathbf{\vert}$ | Pairs per class | ACC | AUC | F1-measure |
| --- | --- | --- | --- | --- | --- | --- | --- | --- | --- | --- |
| EX_nh4_e | Ammonia exchange | 2.5 | limiting | 0.2315 | 29 | 178 | 3996 | 0.803 | 0.858 | 0.807 |
|  |  | 5.0 |  | 0.4629 | 29 | 187 | 3996 | 0.802 | 0.860 | 0.809 |
|  |  | 7.5 |  | 0.6944 | 29 | 190 | 3996 | 0.801 | 0.862 | 0.808 |
|  |  | 9.8 | non-limiting | 0.9073 | 29 | 201 | 3996 | 0.822 | 0.888 | 0.834 |
|  |  | 10.05 |  | 0.9305 | 29 | 201 | 3996 | 0.824 | 0.888 | 0.835 |
|  |  | 10.3 |  | 0.9536 | 29 | 203 | 3996 | 0.825 | 0.888 | 0.836 |
|  |  | 10.61 (baseline) |  | 0.9824 | 29 | 209 | 3996 | 0.825 | 0.889 | 0.835 |
| EX_arg__L_e | L-Arginine exchange | 1.0 | limiting | 0.3703 | 29 | 182 | 3996 | 0.801 | 0.861 | 0.808 |
|  |  | 1.5 |  | 0.5555 | 29 | 189 | 3996 | 0.802 | 0.863 | 0.807 |
|  |  | 2.5 |  | 0.7407 | 29 | 190 | 3996 | 0.801 | 0.862 | 0.809 |
|  |  | 2.45 | non-limiting | 0.9073 | 29 | 191 | 3996 | 0.804 | 0.865 | 0.810 |
|  |  | 2.525 |  | 0.9351 | 29 | 192 | 3996 | 0.826 | 0.887 | 0.834 |
|  |  | 2.6 |  | 0.9629 | 29 | 192 | 3996 | 0.821 | 0.886 | 0.832 |
|  |  | 2.6526 |  | 0.9824 | 29 | 193 | 3996 | 0.823 | 0.886 | 0.834 |
| EX_gln__L_e | L-Glutamine exchange | 2 | limiting | 0.3703 | 28 | 185 | 3871 | 0.810 | 0.867 | 0.818 |
|  |  | 3 |  | 0.5556 | 28 | 191 | 3871 | 0.807 | 0.863 | 0.815 |
|  |  | 4 |  | 0.7407 | 29 | 194 | 3996 | 0.804 | 0.863 | 0.809 |
|  |  | 4.9 | non-limiting | 0.9073 | 29 | 196 | 3996 | 0.802 | 0.863 | 0.810 |
|  |  | 5.05 |  | 0.9351 | 29 | 196 | 3996 | 0.802 | 0.862 | 0.809 |
|  |  | 5.2 |  | 0.9629 | 29 | 196 | 3996 | 0.801 | 0.862 | 0.808 |
|  |  | 5.3052 |  | 0.9824 | 29 | 196 | 3996 | 0.803 | 0.865 | 0.809 |

**Table S7. Evaluation of SARTRE on different phosphorus sources with uptake rates non-limiting and limiting to growth.** Results are obtained by selecting one phosphorus source at a time from sources that are supporting growth. Phosphate is non-limiting between ranges of 0.8528 to 0.9476 mmol gDW^-1^h^-1^ and it becomes limiting with uptake rates below 0.8528 mmol gDW^-1^h^-1^. Phosphonate is non-limiting with uptake rate between 0.8529 and 0.9476 mmol gDW^-1^h^-1^ and becomes limiting with uptake rates below 0.8529 mmol gDW^-1^h^-1^. The number of proteins in all situations is equal to 1365, whose feature size is equal to 1000.

| reaction | reaction name | uptake rate  ($\frac{mmol}{gDW\cdot h})$ | limiting/  non-limiting | biomass flux ($\frac{mmol}{gDW\cdot h})$ | metabolites | $\mathbf{\vert F}^{\mathbf{M}}\mathbf{\vert}$ | Pairs per class | ACC | AUC | F1-measure |
| --- | --- | --- | --- | --- | --- | --- | --- | --- | --- | --- |
| EX_pi_e | Phosphate exchange | 0.25 | limiting | 0.2592 | 29 | 191 | 3996 | 0.804 | 0.858 | 0.809 |
|  |  | 0.50 |  | 0.5183 | 29 | 195 | 3996 | 0.802 | 0.862 | 0.809 |
|  |  | 0.75 |  | 0.7775 | 29 | 199 | 3996 | 0.820 | 0.884 | 0.831 |
|  |  | 0.875 | non-limiting | 0.9071 | 29 | 203 | 3996 | 0.824 | 0.887 | 0.833 |
|  |  | 0.90 |  | 0.9330 | 29 | 203 | 3996 | 0.822 | 0.886 | 0.833 |
|  |  | 0.925 |  | 0.9589 | 29 | 202 | 3996 | 0.821 | 0.885 | 0.833 |
|  |  | 0.9476 (baseline) |  | 0.9824 | 29 | 209 | 3996 | 0.825 | 0.889 | 0.835 |
| EX_ppt_e | Phosphonate exchange | 0.25 | limiting | 0.2592 | 30 | 194 | 5044 | 0.817 | 0.877 | 0.822 |
|  |  | 0.5 |  | 0.5183 | 30 | 199 | 5044 | 0.816 | 0.881 | 0.822 |
|  |  | 0.75 |  | 0.7775 | 30 | 201 | 5044 | 0.818 | 0.882 | 0.823 |
|  |  | 0.875 | non-limiting | 0.9071 | 30 | 205 | 5044 | 0.834 | 0.900 | 0.843 |
|  |  | 0.9 |  | 0.9330 | 30 | 206 | 5044 | 0.833 | 0.894 | 0.842 |
|  |  | 0.925 |  | 0.9589 | 30 | 205 | 5044 | 0.834 | 0.900 | 0.843 |
|  |  | 0.9476 |  | 0.9824 | 30 | 208 | 5044 | 0.833 | 0.898 | 0.842 |

**Table S8. Shared MPIs between GEMs and Datasets.** The number of shared MPIs between datasets and corresponding GEM, the number of interacting pairs (positive class) in datasets, and the percentage of shared pairs relative to all interacting pairs is calculated. By filtering out the shared MPIs, datasets with fewer pairs are created. In consequence, the SARTRE framework is applied to the updated datasets, and new metrics are determined.

| Dataset | Confidence score | GEM | Shared paris | Interacting pairs in GS | Percentage | Filtered pairs in each class | Accuracy | AUC | F1-measure |
| --- | --- | --- | --- | --- | --- | --- | --- | --- | --- |
| Piazza | - | iJO1366 | 141 | 765 | 18.43 | 624 | 0.670 | 0.755 | 0.677 |
| Reznik | - | iJO1366 | 238 | 993 | 23.97 | 755 | 0.736 | 0.804 | 0.740 |
| STITCH-*E. coli* | 150 | iJO1366 | 323 | 7417 | 4.35% | 7094 | 0.762 | 0.827 | 0.772 |
|  | 400 |  | 296 | 3996 | 7.41% | 3700 | 0.828 | 0.893 | 0.838 |
|  | 700 |  | 289 | 2319 | 12.46% | 2030 | 0.826 | 0.891 | 0.836 |
|  | 900 |  | 271 | 866 | 30.59% | 595 | 0.870 | 0.852 | 0.876 |
| STITCH-Yeast | 150 | Yeast-GEM | 465 | 7348 | 6.33% | 6883 | 0.754 | 0.827 | 0.743 |
|  | 400 |  | 430 | 4065 | 10.58% | 3635 | 0.848 | 0.908 | 0.848 |
|  | 700 |  | 401 | 1879 | 21.34% | 1478 | 0.817 | 0.878 | 0.820 |
|  | 900 |  | 379 | 907 | 41.79% | 528 | 0.751 | 0.834 | 0.763 |

## S1. Evaluation of SARTRE with permutation tests

In this section, we evaluate SARTRE by comparing its performance with two null models, in which two permutations as label permutation and feature permutation are applied to the datasets. By considering the original dataset$D=(X,Y)$according to Eq. 12, and the random permutation$\pi$, null distributions are obtained as follows to evaluate the performance of classifier with computed p-value:

First, label permutation is done by applying$\pi$(over$n\times m$elements) on the labels, which results in dataset$D'=(X,\pi(Y))$. This test examines whether the classifier has discovered a real class structure in data and realized a connection between data and labels.

Second, feature permutation is achieved by applying independent permutations to columns of $X$in each class. Here two classes of 0, 1 correspond to non-interaction and interaction classes. Two sub-datasets$X_{0}$and$X_{1}$contain separate entries of each class. Then independent permutations$\pi_{0}$and$\pi_{1}$are applied to the columns of sub-datasets$X_{0}$and$X_{1}$, respectively,  which results in permuted sub-datasets$X_{0}'$and$X_{1}'$. A randomized version of the dataset $D'=(X',Y)$ is constructed, in which$X'=(X_{0}',X_{1}')$.This test assesses whether the classifier exploits dependency between features.

We performed label and feature permutations on SARTRE to illustrate whether the extracted features are valuable, and we performed permutation tests on our datasets. It should be noted that first, 10-fold cross validation is applied to a dataset and random undersampling is executed only on the train set and the distribution of the test folds are not manipulated. The p-values (<0.01) shown in Table S9 indicate that the classifier does not show performance expected at random, and the extracted features of metabolites and proteins are informative.

**Table S9.** **Evaluation of SARTRE with permutation tests.** Label permutation and feature permutation is applied to the original datasets and considered as an input of the random forest classifier and AUC metric is employed for evaluation. AUC of the classifier with 10-fold cross validation for original datasets and constructed null distributions are presented. P-values for rejection of the null hypothesis is shown for each of the permuted datasets beside the AUC metric in parenthesis.

| Dataset | Confidence score | Original | Label permutation | Feature permutation |
| --- | --- | --- | --- | --- |
| Piazza | - | 0.680 | 0.501 (1.697e-7) | 0.504 (8.97e-17) |
| Reznik | - | 0.726 | 0.508 (3.72e-13) | 0.606 (9.46e-7) |
| STITCH-*E. coli* | 150 | 0.770 | 0.479 (4.79e-18) | 0.501 (3.88e-21) |
|  | 400 | 0.824 | 0.488 (6.147e-14) | 0.478 (1.913e-13) |
|  | 700 | 0.816 | 0.509 (2.314e-11) | 0.480 (1.220e-14) |
|  | 900 | 0.774 | 0.521 (1.728e-13) | 0.498 (9.910e-19) |
| STITCH-Yeast | 150 | 0.760 | 0.508 (2.58e-18) | 0.549 (1.207e-8) |
|  | 400 | 0.841 | 0.485 (2.00e-17) | 0.567 (8.67 e^-7^) |
|  | 700 | 0.805 | 0.504 (3.99e-16) | 0.542 (7.70e-8) |
|  | 900 | 0.800 | 0.506 (1.260e-14) | 0.559 (1.080e-6) |
